# Emerging roles of the cGAS/STING pathway in cardiovascular diseases

**DOI:** 10.1172/JCI204551

**Published:** 2026-07-01

**Authors:** Wataru Saitoh, Yasutomi Higashikuni, Oyunbileg Bavuu, Masataka Sata, Daiju Fukuda

**Affiliations:** 1 Department of Cardiovascular Medicine, Osaka Metropolitan University Graduate School of Medicine, Osaka, Japan.; 2 Division of Cardiovascular and Genetic Research, Center for Molecular Medicine, and; 3 Department of Cardiovascular Medicine, Jichi Medical University, Tochigi, Japan.; 4 Department of Cardiovascular Medicine, The University of Tokyo, Tokyo, Japan.; 5 Department of Cardiovascular Medicine, Tokushima University Graduate School, Tokushima, Japan.

## Abstract

Cardiovascular diseases (CVDs) remain the leading cause of mortality and morbidity worldwide, highlighting the need for novel therapeutic approaches. Inflammation plays a key role in CVD pathogenesis, and accumulating evidence has implicated the cyclic GMP-AMP synthase/stimulator of IFN genes (cGAS/STING) pathway in this process. The cGAS/STING pathway recognizes both non-self- and self-DNA, including mitochondrial and nuclear DNA, to activate its downstream proinflammatory signaling molecules, including TANK-binding kinase 1, IFN regulatory factor 3, and NF-κB. Various pathological stressors have been shown to induce self-DNA release into the cytosol and bloodstream from damaged cells in the cardiovascular system, indicating that circulating cell-free DNA is a useful biomarker of CVDs; however, how this contributes to the inflammatory signaling, cell death, and fibrosis that characterize CVDs remains unclear. Here, we discuss the current understanding on the roles of self-DNA and the cGAS/STING pathway in the pathophysiology of CVDs and the therapeutic potential of targeting this pathway.

## Introduction

Cardiovascular diseases (CVDs) represent a wide array of disorders affecting the heart and blood vessels, including atherosclerosis, hypertension, pulmonary hypertension (PH), aortic aneurysm and dissection (AAD), myocardial infarction (MI), hypertensive heart disease, valvular heart disease, cardiomyopathy, myocarditis, and atrial fibrillation (AF). Despite progress in medical therapies, CVDs remain the leading cause of mortality and morbidity worldwide (1). The prevalence of CVDs, especially in the aging population, is expected to rise in the next few decades (2). To address this global burden, novel therapeutic strategies need to be developed.

**Figure 1:**
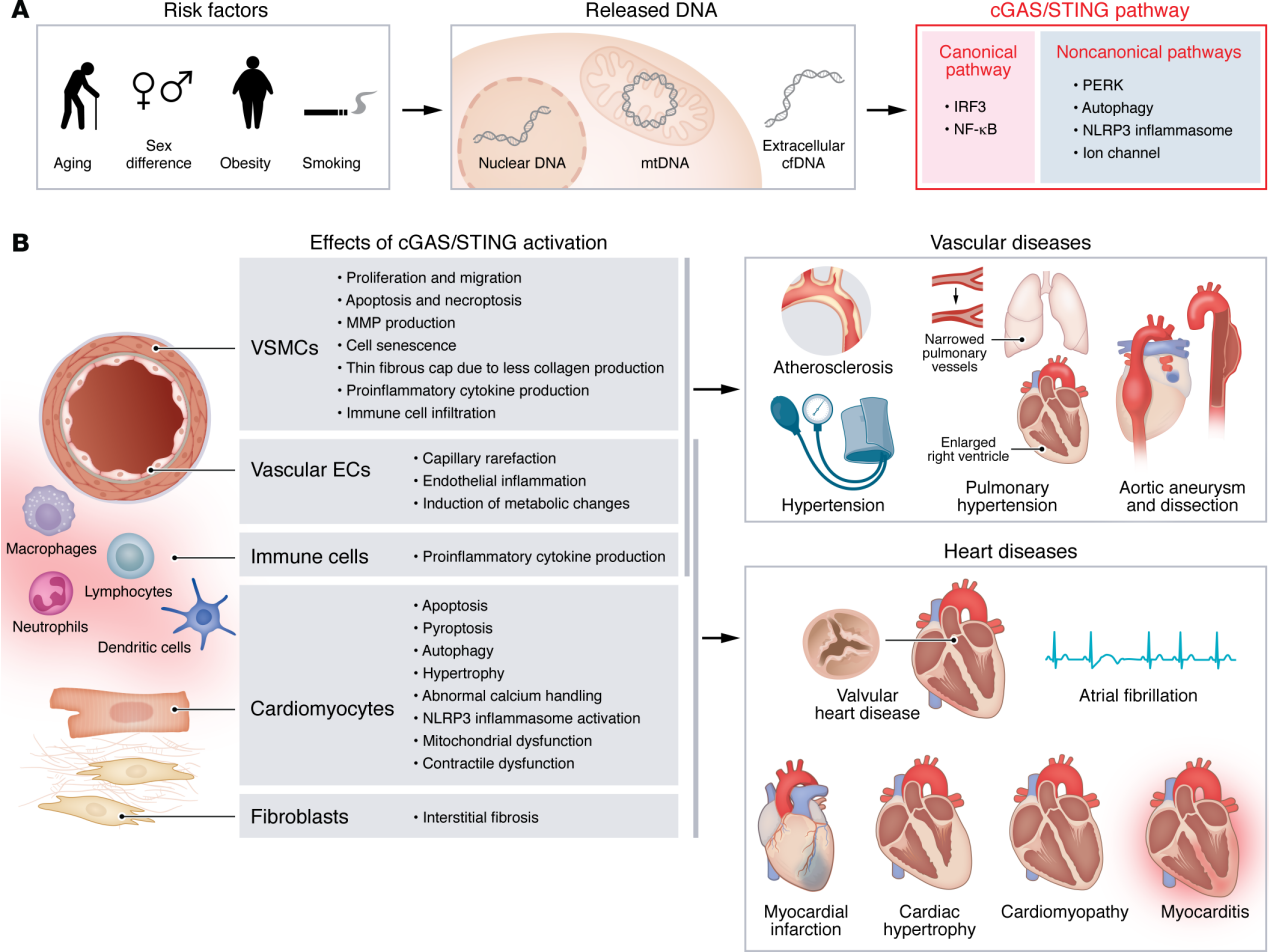
The cGAS/STING pathway and cardiovascular diseases. (**A**) Risk factors associated with cardiovascular disease — such as aging, sex differences, obesity, and smoking — contribute to the accumulation of intracellular nuclear DNA and mtDNA and extracellular cell-free DNA (cfDNA), which activate both canonical and noncanonical cGAS/STING signaling. (**B**) Activation of the cGAS/STING pathway modulates the function of vascular and cardiac cells, including vascular smooth muscle cells (VSMCs), vascular endothelial cells (ECs), immune cells, cardiomyocytes, and fibroblasts. This causes functional and structural changes of the blood vessels and heart, contributing to the pathophysiology of cardiovascular diseases, including atherosclerosis, hypertension, pulmonary hypertension, aortic aneurysm and dissection, myocardial infarction, myocardial ischemia/reperfusion injury, hypertensive heart disease, valvular heart disease, cardiomyopathy, myocarditis, and atrial fibrillation.

The cyclic GMP-AMP synthase/stimulator of IFN genes (cGAS/STING) pathway is a component of the innate immune system that senses cytosolic double-stranded DNA (dsDNA) to trigger proinflammatory gene expression. Originally identified for its role in host defense against viral and bacterial infection through recognition of non-self-DNA, the cGAS/STING pathway has also been implicated in sterile inflammation by detecting self-DNA released from damaged or stressed cells (Figure 1) (3–6). Here, we describe the emerging roles of the cGAS/STING pathway in the pathophysiology of CVDs and discuss therapeutic strategies targeting the pathway.

## Overview of the cGAS/STING pathway

The cGAS/STING pathway links non-self- or self-dsDNA sensing to activation of proinflammatory transcription factors (Figure 1). cGAS, also known as MB21D1, is a member of the nucleotidyltransferase (NTase) enzyme family that detects cytosolic dsDNA in a sequence-independent manner (7–10). Upon binding to dsDNA, cGAS dimerizes and undergoes conformational changes that lead to the opening of a catalytic nucleotide binding pocket. Activated cGAS catalyzes the formation of the second messenger cyclic GMP-AMP (cGAMP) from ATP and GTP (10–14). The synthesized cGAMP binds to an active pocket site of STING, triggering its downstream signaling.

STING is a dimeric transmembrane adaptor protein localized on the ER, with its ligand-binding domain exposed to cytosol (10, 15–17). Upon binding to cGAMP, STING undergoes conformational changes to form a stable tetramer or higher-order oligomer that translocates from the ER to the Golgi apparatus, where STING recruits TANK-binding kinase 1 (TBK1) to promote the phosphorylation of TBK1 and STING (17, 18). Phosphorylated TBK1 promotes the phosphorylation and nuclear trafficking of downstream transcription factors, such as IFN regulatory factor 3 (IRF3) and STAT6 (7, 8, 17, 19). IRF3 induces expression of IFN-I and subsequent expression of IFN-stimulated genes (ISGs), while STAT6 induces chemokine expression (20, 21). STING can also activate NF-κB in both TBK1-dependent and -independent manners, which further promotes the expression of proinflammatory genes (10, 15, 16, 22).

## DNA recognized by the cGAS/STING pathway

cGAS recognizes dsDNA in the cytosol from both extracellular and intracellular origins (Figure 1) (7). DNA fragments from microorganisms, viruses, dead cells (cell-free DNA), and extracellular vesicles can enter the cytosol by endocytosis, phagocytosis, or transmembrane diffusion (23–26). DNA fragments of intracellular origin include DNA released from mitochondria (mtDNA), chromatin fragments, and damaged DNA packaged into micronuclei in the nucleus (10). Although agnostic to the specific sequence, cGAS interacts with dsDNA in a nucleotide length–dependent manner (27–30). It has been reported that dsDNA fragments longer than 20 base pairs can bind cGAS but dsDNA fragments longer than 40 base pairs form a more stable complex for cGAS activation (29, 30).

## cGAS/STING in the pathophysiology of vascular diseases

### Atherosclerosis.

Atherosclerosis is the thickening and hardening of the medium- and large-sized arteries due to a buildup of plaque in the subendothelium that consists of cholesterol, fatty acids, calcium, fibrin, blood cells, cellular waste products, and other substances in the blood. A large body of evidence now indicates that inflammation fundamentally contributes to the pathogenesis of atherosclerosis and accompanying thromboembolic events due to plaque rupture or erosion and that the cGAS/STING pathway plays important roles in producing that inflammation (Figure 1 and Tables 1 and 2). Upregulation of STING is observed in human atherosclerotic lesions (Table 1) (31, 32). In a mouse model of diabetes, genetic deletion and pharmacologic inhibition of STING ameliorate aortic endothelial dysfunction (33). Genetic disruption of IRF3 in *Apoe^–/–^* mice resulted in reduced lipid burden and macrophage infiltration in atherosclerotic plaques (34).

In the arterial walls, the cGAS/STING pathway can be activated in various cell types, including vascular endothelial cells (ECs), vascular smooth muscle cells (VSMCs) and immune cells. Dong et al. reported that STING was upregulated in vascular ECs in *Apoe^–/–^* mice fed a high-fat diet, and EC-specific deletion of STING in high-fat diet–fed *Apoe^–/–^* mice resulted in a meaningful reduction in atherosclerotic plaques (32). Mechanistically, mtDNA, which is released into the cytoplasm of vascular ECs in response to low oscillatory shear stress, was suggested to activate the cGAS/STING pathway, leading to EC senescence and dysfunction and promoting atherogenesis. Oxidized LDL also induces mtDNA release in vascular ECs in *Apoe^–/–^* mice fed a Western diet, further contributing to STING activation and atherogenesis (35). Interestingly, activation of the cGAS/STING pathway by mtDNA was reported to contribute to NLRP3 inflammasome activation and subsequent pyroptosis in vascular ECs (36, 37). In vitro, treatment of human umbilical vein ECs with mtDNA or cGAMP increased inflammatory molecule expression and decreased endothelial NOS phosphorylation, partially through the cGAS/STING pathway (33).

Cigarette smoke extract can induce DNA damage in both the nucleus and mitochondria in human ECs, leading to IL-6 upregulation through activation of the cGAS/STING pathway (38). In addition, the cGAS/STING pathway was shown to activate the noncanonical effector PKR-like ER kinase (PERK), which causes vascular EC dysfunction through epigenetic alterations (39). Furthermore, Liu et al. demonstrated that IRF3 upregulation in vascular ECs contributes to plaque vulnerability through adhesion molecule induction in *Apoe^–/–^* mice (34). Collectively, these findings indicate the diverse contribution of the cGAS/STING pathway to vascular EC function. However, it is important to note that ECs are heterogenous, with different populations having differing roles in atherosclerosis (40), and the role of the cGAS/STING pathway in EC heterogeneity remains to be elucidated. In addition, the effect of the cGAS/STING pathway on vascular ECs in the peripheral arteries and small vessels needs to be clarified.

A role for the cGAS/STING pathway in atherosclerotic macrophages has also been reported. In *Apoe^–/–^* mice fed a Western diet, genetic deletion of STING ameliorated atherosclerosis, with decreased macrophage infiltration and proinflammatory cytokine expression in the aorta, whereas restoration of STING expression in bone marrow–derived cells in *Sting^–/–^;Apoe^–/–^* mice promoted atherogenesis with increased proinflammatory cytokine expression and TBK1 phosphorylation in the aorta (31). The release of mtDNA and subsequent activation of the cGAS/STING pathway have also been reported in oxidized LDL-treated macrophages (41, 42).

In VSMCs, the cGAS/STING pathway is implicated in senescence. Senescent VSMCs produce less collagen and more proinflammatory cytokines, which results in a thin fibrous cap and immune cell infiltration and promotes plaque vulnerability (43, 44). Bi et al. reported that, in *Apoe^–/–^* mice with chronic kidney disease, mtDNA released from damaged mitochondria activates the cGAS/STING pathway, resulting in IFN-I production, which causes VSMC senescence and phenotypic switching and promotes plaque vulnerability (43). Uryga et al. demonstrated that telomere-derived damaged DNA induces cellular senescence and proinflammatory cytokine expression in VSMCs through the cGAS/STING pathway, which leads to accelerated atherogenic changes after vascular injury (44).

### Hypertension.

Hypertension is the most prevalent CVD, in which systemic BP is persistently elevated. Mechanisms of hypertension include high-salt intake, activation of the renin-angiotensin-aldosterone system, increased sympathetic drive, and impaired nitric oxide bioavailability. Accumulating evidence implicates vascular inflammation in the initiation and maintenance of hypertension (45).

A recent study highlighted an important role of the cGAS/STING pathway in the development of hypertension (Figure 1 and Table 3) (46). Li et al. demonstrated that, in mice expressing a human risk variant of apolipoprotein L1 associated with hypertension, STING activation in vascular ECs was associated with hypertension, which promoted endothelin 1 production (46). EC-specific STING deletion markedly attenuated hypertension and subsequent vascular remodeling with suppressed endothelin 1 expression, demonstrating a causal role of STING activation in hypertension.

Chronobiological rhythms regulate BP and its variability (47, 48), and disruption of those rhythms has been linked to the pathophysiology of hypertension (47, 48). A recent study reported that disruption of chronobiological rhythm activates the cGAS/STING pathway and causes cell senescence in macrophages (49). Since innate immune cells, including macrophages, have been implicated in the pathophysiology of hypertension (50, 51), the cGAS/STING pathway might link disrupted chronobiological rhythms to hypertension. Further studies are needed to clarify the role of the cGAS/STING pathway in circadian control of BP and its variability.

### PH.

PH is a condition of high BP in the arteries of the lungs (52). Pham et al. observed STING upregulation in the lung of patients with PH (Table 1) (53, 54). In rodent models of PH, genetic disruption or pharmacologic inhibition of STING has been shown to improve pulmonary vascular remodeling and hypertension (Table 3) (54–58). Mechanistically, the cGAS/STING pathway induces VSMC senescence in the pulmonary arteries through NF-κB activation (54–57). Wu et al. showed that STING activates the NLRP3 inflammasome in macrophages to induce proliferation of VSMCs through proinflammatory cytokine production (59). In addition, the cGAS/STING pathway is implicated in immune cell dysfunction in PH. Pham et al. demonstrated that genetic deletion or pharmacologic inhibition of STING increases VEGF in CD11b^+^ and CD11c^+^ myeloid cells, which might contribute to protection against PH in an IFN signaling–independent manner (53). Interestingly, Pham et al. also reported that STING expression in myeloid cells might prevent severe PH by suppressing expression of PD-L1, suggesting diverse roles of STING in myeloid cells (54).

### AAD.

An aortic aneurysm is a dilatation of the aorta to greater than 1.5 times the original size due to structural weakening of the aortic wall. An aortic dissection is a tear between the intima and media of the aortic wall. AADs are lethal vascular conditions, characterized by progressive VSMC loss and extracellular matrix degradation, which can lead to aortic rupture or severe ischemia due to arterial occlusions. Persistent vascular inflammation has been implicated in the pathogenesis of AAD (60).

Recent studies have demonstrated that the cGAS/STING pathway plays important roles in the underlying mechanisms of AAD (Figure 1 and Table 4). Luo et al. reported marked upregulation of STING, TBK1, and IRF3 expression and phosphorylation in human sporadic AAD tissues (Table 1) (61). In a murine model of sporadic AAD induced by a combination of a high-fat diet and high-dose angiotensin II infusion, STING-deficient mice exhibited marked reductions in aortic enlargement, dissection, and rupture. Nuclear and mitochondrial DNA damage in VSMCs, and the subsequent leakage of DNA to the cytosol and extracellular space, activates STING in neighboring VSMCs and macrophages, leading to apoptotic and necroptotic cell death. Using single-cell transcriptome and transposase-accessible chromatin analyses, Chakraborty et al. revealed that activation of STING and IRF3 induces epigenetic modification and chromatin remodeling in VSMCs in the aorta of angiotensin II–infused mice, which resulted in VSMC phenotypic alterations through suppression of contractile genes and induction of proinflammatory genes (62). These epigenetic and phenotypic changes in VSMCs were prevented in *Sting^–/–^* mice.

## cGAS/STING in the pathophysiology of heart diseases

Heart diseases, including MI, hypertensive heart disease, valvular heart disease, cardiomyopathy, myocarditis, and AF all cause cardiac remodeling that can lead to heart failure (HF) (63, 64). A growing body of experimental and clinical evidence now demonstrates that inflammation plays a fundamental role in the pathophysiology of HF (65), although the underlying mechanisms remain largely unknown. Recent studies have provided evidence supporting involvement of the cGAS/STING pathway in various heart diseases and HF (17) (Figure 1 and Tables 1–4).

### MI.

MI is a disease characterized by myocardial cell death due to the interruption of blood flow in the coronary arteries to the corresponding region of the myocardium. Atherosclerosis is a main cause of MI. Following occlusion of the coronary arteries, intense sterile inflammation and immune cell infiltration, triggered by molecules released from damaged or dead cells, are initiated to digest and clear damaged cells and extracellular matrix (66). Subsequently, reparative pathways promote myofibroblast proliferation, neovascularization, resolution of inflammation, and scar formation for tissue repair. After the reparative phase, the heart continues to undergo structural and functional changes that lead to chamber dilatation and contractile dysfunction and exacerbate HF. Inflammation has been implicated not only in the reparative phase, but also in chronic cardiac remodeling after MI.

The cGAS/STING pathway has been implicated in the initiation of cardiac inflammation and the clearance of necrotic myocardium and tissue repair after MI (Figure 1). Upregulation of STING and IRF3 in the heart was observed in the reparative phase of murine hearts after MI (67, 68). King et al. found that genetic disruption of IRF3 or cGAS improved survival rate and cardiac remodeling after MI (67). Mechanistically, the cGAS/STING/IRF3 pathway is activated in macrophages that recognize dsDNA released from dying cells in the infarcted region. Subsequently, the signaling induces the production of IFN-I and ISGs, which impairs tissue repair and deteriorates cardiac remodeling after MI by promoting the expression of proinflammatory cytokines and chemokines and recruitment of leukocytes in the infarcted heart. Cao et al. also reported that the cGAS/STING pathway is essential for maintaining proinflammatory macrophages in the murine heart after MI through inducible NOS upregulation (68). Furthermore, Zhu et al. demonstrated that the cGAS/STING pathway was associated with neutrophil production and activation, although cGAS deficiency in neutrophils did not affect neutrophil extracellular trap (NET) formation after MI (69). Neutrophil-specific deletion of cGAS improved survival after MI in mice, although it did not affect post-MI cardiac remodeling.

### Myocardial ischemia/reperfusion injury.

Myocardial ischemia/reperfusion injury is myocardial damage caused by temporary interruption and subsequent restoration of blood flow to the heart (70). ROS, generated by the reactivated electron transport chain and other sources, play important roles in the pathogenesis of myocardial ischemia/reperfusion injury (70). ROS cause cardiomyocyte damage and cardiac dysfunction by inducing apoptosis, autophagy, and proinflammatory responses. Damage-associated molecules released from necrotic cardiac cells, including mitochondrial and nuclear DNA, also contribute to activation of proinflammatory responses (71).

Activation of the cGAS/STING pathway and accumulation of mtDNA in the cytosol have been reported in cardiac tissues of diabetic mice exposed to myocardial ischemia/reperfusion injury. Pharmacological inhibition of STING attenuated myocardial damage in this model (72). Zhang et al. reported that mitochondrial components, released from cardiomyocytes through extracellular vesicles, might be internalized by cardiac fibroblasts and promote myocardial fibrosis via fibroblast activation and proliferation (73). These findings suggest a potential role of the cGAS/STING pathway in the pathophysiology of myocardial ischemia/reperfusion injury, although further studies are necessary to establish its relevance in the human disease.

### Hypertensive heart disease.

Hypertensive heart disease is a condition in which cardiac structural and functional changes are attributable to systemic hypertension. Cardiac hypertrophy initially occurs as an adaptive response to pressure overload to maintain cardiac function (63). However, chronic stress responses cause contractile dysfunction and HF (64). Inflammation has been implicated in the pathophysiology of both adaptive hypertrophy and HF (74, 75).

The roles of the cGAS/STING pathway in hypertensive heart disease have been investigated by using a murine model of pressure overload-induced cardiac hypertrophy and HF. Upregulation of cGAS and STING has been reported in the murine heart subjected to pressure overload as well as in the human heart exhibiting cardiac hypertrophy (Table 1) (76–80). Hu et al. demonstrated that cGAS knockdown in cardiomyocytes improves survival rate and ameliorates cardiac hypertrophy, contractile dysfunction, interstitial fibrosis, immune cell infiltration, proinflammatory cytokine expression, and cardiomyocyte apoptosis during pressure overload (76). Zhang et al. reported that genetic deletion of STING resulted in improvement of cardiac hypertrophy, interstitial fibrosis, and contractile dysfunction with suppressed macrophage infiltration, IFN-I–mediated inflammation, and ER stress response in pressure-overloaded hearts (77). Guo et al. showed that upregulation of inducible NOS during pressure overload contributes to mtDNA release in cardiomyocytes, which promotes adverse remodeling through activation of the cGAS/STING/IRF3 pathway in mice (78). Brassington et al. reported that activation of the cGAS/STING pathway in cardiomyocytes might be involved in CD8^+^ T cell activation and macrophage recruitment, which induce cardiomyocyte apoptosis and interstitial fibrosis, respectively (81). Sanders et al. demonstrated that STING activation in cardiac ECs induces IL-6 secretion and HF during pressure overload in mice (82). Interestingly, transgenic mice with cardiomyocyte-specific overexpression of STING showed attenuated adverse remodeling and contractile dysfunction in response to pressure overload by inhibiting autophagy (79), while cardiomyocyte-specific activation of STING, using a constitutively active mutant, led to cardiac hypertrophy and HF in mice (80). How STING contributes to the adaptive and maladaptive mechanisms in hypertensive heart disease remains to be elucidated.

### Valvular heart disease.

Valvular heart disease is a structural or functional abnormality of cardiac valves that affects blood flow and mechanical stress on the heart. Progression of aortic valve calcification results in aortic stenosis (83). Hu et al. reported increased STING and its phosphorylation in calcified human valves compared with noncalcified valves (Table 1) (84). Pharmacological inhibition of STING suppressed calcium nodule formation and alkaline phosphatase activity in human aortic valve interstitial cells (HAVICs) treated with osteogenic medium, whereas treatment with a STING agonist promoted these changes. Genetic disruption or pharmacologic inhibition of STING also mitigated aortic valve calcification in mice. Mechanistically, mtDNA leakage through downregulation of calcium/calmodulin-dependent protein kinase 1 in activated HAVICs stimulates the cGAS/STING pathway, which leads to upregulation of alkaline phosphatase and runt-related transcription factor 2, the essential regulators of calcification, through TBK1 and NF-κB activation (84–86).

### Cardiomyopathy.

Cardiomyopathies are diseases of the heart muscle characterized by morphologically and functionally abnormal myocardium due to various causes, in the absence of coronary artery disease, hypertension, valvular disease, and congenital heart disease sufficient to cause the observed myocardial abnormality (87, 88). STING upregulation has been reported in human heart samples of patients with dilated and hypertrophic cardiomyopathy (HCM) (77), suggesting the involvement of the cGAS/STING pathway (Table 1).

Dilated cardiomyopathy is the most common form of cardiomyopathy, characterized by chamber dilation and dysfunction. Patients carrying a homozygous missense mutation in the nuclear lamina-associated inner nuclear membrane protein LEMD2 develop dilated cardiomyopathy (89). Using a knockin mouse model of the *LEMD2* disease mutation and corresponding cell model (90), Chen et al. demonstrated that this mutation impairs nuclear envelope rupture repair capacity. The resulting accumulation of DNA in the cytoplasm activates cGAS/STING and promotes myocardial cellular senescence. Mutations in the *LMNA* gene, encoding the nuclear envelope protein lamin A/C, cause dilated cardiomyopathy (91). Cheedipudi et al. showed that genetic disruption of cGAS attenuated the dilated cardiomyopathy phenotype in *Lmna*-deficient mice (92). On the other hand, En et al. reported that the cGAS/STING pathway is not activated in adult mice with cardiomyocyte-specific *Lmna* deletion (93). Further investigation will be necessary to clarify the role of the cGAS/STING pathway in lamin-related dilated cardiomyopathy.

HCM is characterized by thickening of the heart muscle without pressure overload. The cGAS/STING/IFN pathway is implicated in the pathophysiology of sporadic HCM caused by a somatic loss-of-function mutation in *NAP1L1*, which encodes a member of the nucleosome assembly protein family (94). Lv et al. showed that in vivo overexpression of this variant in cardiomyocytes exacerbated angiotensin II–induced cardiac hypertrophy with activation of the cGAS/STING pathway in mice, while pharmacological inhibition of STING and IFN receptor reversed the cardiac phenotype (94). Mechanistically, this mutation causes destabilization of nucleosome formation and DNA leakage into the cytoplasm, which accelerates proinflammatory responses and cardiac hypertrophy.

Diabetic cardiomyopathy is characterized by lipid accumulation, mitochondrial dysfunction, inflammation, and fibrosis (95). Activation of the cGAS/STING pathway and the NLRP3 inflammasome have been observed in murine diabetic hearts (96–98). Yan et al. demonstrated that *Sting* knockdown in cardiomyocytes improved diabetes-induced cardiac hypertrophy and contractile dysfunction with suppressed pyroptosis and inflammatory responses (96). Mechanistically, palmitic acid–induced lipotoxicity activated the cGAS/STING pathway through mitochondrial oxidative damage and mtDNA release into the cytosol in cardiomyocytes, which promoted pyroptosis and proinflammatory cytokine production in an NLRP3 inflammasome-dependent manner, suggesting crosstalk between the cGAS/STING pathway and NLRP3 inflammasome activation (96–98).

Chronic kidney disease has been shown to contribute to the pathophysiology of cardiomyopathy (99). Han et al. reported the activation of the cGAS/STING pathway in murine hearts in a chronic kidney disease model, which was ameliorated by cardiomyocyte-specific genetic disruption of STING (100). Mitochondrial oxidative stress was implicated in activation of the cGAS/STING pathway in this setting.

Anthracycline chemotherapeutics for the treatment of cancer, such as doxorubicin, have been shown to cause cardiotoxicity with a poor prognosis (101, 102), and chronic doxorubicin cardiomyopathy has been associated with activation of the cGAS/STING pathway in murine hearts (103–105). Luo et al. demonstrated that EC-specific genetic deletion of STING prevented doxorubicin cardiomyopathy and EC dysfunction (103). Mechanistically, activation of the cGAS/STING pathway in cardiac ECs by doxorubicin caused mitochondrial dysfunction through a reduction of intracellular NAD levels. Another chemotherapeutic, cisplatin, has also been reported to have cardiotoxic effects (106) and shown to activate the cGAS/STING pathway in murine hearts (107). Wang et al. demonstrated that genetic disruption of STING attenuated cisplatin-induced cardiotoxicity in mice (107).

Immune checkpoint inhibitors are another group of cardiotoxic anticancer drugs (108). Cao et al. demonstrated that anti–PD-1 antibody activates the cGAS/STING pathway in macrophages with M1 polarization (109). Pharmacological inhibition of STING ameliorated cardiac injury induced by anti–PD-1 antibody in mice, although the mechanism remains unelucidated.

### Myocarditis.

Myocarditis is an inflammatory disease of the heart, characterized by inflammatory cell infiltration and myocardial injury, which can be caused by infections, immune systemic activation, and exposure to drugs (110). The cGAS/STING pathway has been implicated in the pathophysiology of various types of myocarditis (111–116). Qin et al. reported that the cGAS/STING pathway contributes to cardiac inflammation by coxsackievirus B3 (CVB3) infection (111). Genetic disruption of STING suppressed CVB3-induced proinflammatory cytokine expression in mice, which led to higher survival rates. CVB3-infected cardiomyocytes release mtDNA into the extracellular space, which activates STING signaling in cardiac macrophages. An important role of cGAS in macrophages was also reported in Chagas disease, which can cause myocarditis by *Trypanosoma cruzi* infection (112). Choudhuri et al. reported that extracellular vesicles containing oxidized DNA are released from infected cardiac cells, which activates macrophages through cGAS signaling (112). Li et al. demonstrated that STING is activated in a murine sepsis-induced cardiac injury model induced by LPS injection (113). In addition, genetic deletion of STING was shown to improve survival and cardiac function in this model, associated with suppressed cardiac inflammation and cardiomyocyte apoptosis and pyroptosis.

The expression of STING and single-stranded DNA accumulation in macrophages were also observed in human heart samples of patients with autoimmune myocarditis (114). Mice with genetic disruption of TREX1 or DNase II have been reported to exhibit autoimmune myocarditis with elevated expression of ISGs (115, 116). Gao et al. demonstrated that genetic ablation of cGAS in these murine models rescued the cardiac phenotypes (115). In another experimental autoimmune myocarditis model using subcutaneous injections of cardiac myosin heavy chain α peptides, Hua et al. showed that pharmacological inhibition of STING ameliorated myocarditis with suppressed proinflammatory responses in macrophages (114). Further studies will be necessary to clarify mechanistic insight more in detail.

### AF.

AF is the most common arrhythmia, characterized by irregular beating of the atrial chambers of the heart (117). Upregulation and activation of the cGAS/STING pathway have been reported in the atria of rodent models of diabetes and obesity (118, 119). Diabetic mice or obese rats show increased susceptibility to AF induced by rapid atrial pacing, but this phenotype was ameliorated by cardiomyocyte-specific knockdown of STING (118, 119).

STING activation is associated with atrial fibrosis, cardiomyocyte apoptosis, and abnormal calcium handling (118). In the atria of obese rats, expression levels of inositol 1,4,5-trisphosphate receptor type 1 (IP3R1) on the ER and voltage-dependent anion channel protein 1 (VDAC1) on the mitochondrial outer membrane are upregulated, resulting in excessive calcium transfer from the ER to the mitochondria (120). STING knockdown mitigated these structural and molecular changes. Interestingly, mitochondrial calcium overload mediated by IP3R1 and VDAC1 was reported to promote the leakage of mtDNA into the cytoplasm, leading to activation of cGAS (121). This finding suggests that the cGAS/STING pathway contributes to a vicious cycle in the pathophysiology of AF.

## Targeting cGAS/STING for the treatment of CVDs

Pharmacological inhibitors of the cGAS/STING pathway have been developed, such as cGAS inhibitors, STING inhibitors, and TBK1 inhibitors (Table 5), and have been under clinical investigation in autoimmune disease and cancer (122, 123). VENT-03, an oral cGAS inhibitor, was shown to be safe and well-tolerated in healthy volunteers and is now in a phase IIa trial for the treatment of systemic lupus erythematosus (124). In cancer, nucleotide-based cGAMP analogs, which aim to exploit cGAS/STING-dependent antitumor immunity, have been tested, although trials have not yet shown therapeutic benefit (125). In infectious disease, modulation of cGAS/STING to either augment host defense or mitigate excessive inflammation has been under exploration (126). Collectively, these attempts provide important conceptual and translational frameworks for applying cGAS/STING-targeted therapies to CVDs. Here, we describe evidence of the therapeutic potential of these agents as demonstrated in animal models of CVDs.

### cGAS inhibitors.

Three types of cGAS inhibitors have been developed: inhibitors of cGAS enzymatic activity, inhibitors of cGAS binding to dsDNA, and targeted modulators of cGAS by acetylation. RU.521 is a competitive inhibitor of cGAS that binds to the active site of cGAS and inhibits its interaction with ATP and GTP (127). RU.521 has been shown to be effective in the treatment of atherosclerosis, myocardial ischemia/reperfusion injury, and myocarditis in mice (36, 114, 128, 129). X6 and antimalarial drugs, which prevent the formation of the cGAS-dsDNA complex, were shown to have a beneficial effect in murine autoimmune myocarditis (116). Interestingly, the NSAID aspirin acetylates cGAS, which inhibits cGAS activation and improves autoimmune myocarditis in mice (130). Further studies are needed to clarify therapeutic potentials of these agents in human disease.

### STING inhibitors.

Nitrofuran derivatives, such as C-176, C-178, and H-151, effectively inhibit STING signaling by binding to Cys91, which results in nitroalkylation of STING’s palmitoylation site (131). These compounds have been shown to improve atherosclerosis, MI, myocardial ischemia/reperfusion injury, hypertensive heart disease, cardiomyopathy, and myocarditis in mice (31, 36, 43, 81, 100, 114, 128, 132, 133). Astin C and SN-011 competitively block interaction between STING and cyclic dinucleotides. These compounds showed protective effects against autoimmune myocarditis in mice (134, 135).

### TBK1 inhibitors.

Several kinase inhibitors, including K252a, dovitinib, and oxindole 91, have been shown to inhibit TBK1 by activity-based screens of compound libraries, although they are not TBK1-specific inhibitors (136). These compounds prevent autophosphorylation of TBK1 and IκB kinase ε (IKKε) at the Ser172 site through hydrogen bond network formation. Amlexanox is another TBK1 inhibitor that prevents its phosphorylation at the Ser366 site, and it also has an inhibitory effect on IKKε. Amlexanox is currently used for the treatment of recurrent aphthous ulcers and bronchial asthma. Several studies have shown that amlexanox mitigates atherosclerosis and AAD in mice (31, 61). Further investigation will be needed to determine the potential of TBK1 inhibitors in the treatment of CVDs.

## Conclusions and remaining questions

The cGAS/STING pathway is a pivotal regulator of the innate immune system that functions through the sensing of both non-self- and self-DNA. Although indispensable for host defense, uncontrolled activation of the cGAS/STING pathway by self-DNA can drive chronic sterile inflammation and contribute to the development of a wide spectrum of diseases, including CVDs, which causes further cell and tissue damage to release self-DNA. Many questions remain regarding the contribution of cGAS/STING to CVD pathogenesis, and it will be important to address these in order to successfully translate therapies targeting the pathway.

Lifestyle factors, including smoking and obesity, have been reported to cause self-DNA release from damaged cells and tissues, which exacerbates inflammation via the cGAS/STING pathway (137). For example, Ueda et al. reported that in atherosclerotic patients, the serum level of circulating cell-free DNA was higher in smokers than in nonsmokers (38). It is possible that lifestyle modifications that can improve clinical outcomes of CVDs act in part by preventing the DNA release that activates the cGAS/STING pathway.

Aging is a risk factor for CVDs, and the effects may in part be mediated by cGAS/STING pathway activation. For example, levels of circulating mtDNA increase with age (138, 139), and nuclear integrity is impaired with age due to dysfunction of Yes-associated protein and transcriptional coactivator with PDZ-binding motif (140). Furthermore, levels of the cytosolic exonuclease TREX1 decrease in the kidney in aged rodents (141). These changes could result in cGAS/STING activation and drive senescence (142–144). Biological sex also affects CVD risk, and sex differences in protein expression profiles related to the cGAS/STING pathway have been reported (141, 145, 146). For example, activity of the cGAS/STING pathway is higher in the kidneys of male rats than in female rats, and this difference becomes more prominent with age (141). Such differences might contribute to the more pronounced age-related increase in the cumulative incidence of CVDs in men compared with women (147).

Many immune cell types contribute to the pathogenesis of CVDs, but the role of cGAS/STING in those populations is not fully elucidated. For example, granulocytes can form NETs to propagate inflammation and thrombosis in atherosclerotic plaques (148, 149), and the DNA in those NETs can be recognized by cGAS (150). However, whether the cGAS/STING pathway in these cells plays a role in the pathophysiology of CVDs needs further investigation.

In addition to the canonical cGAS/STING pathway that activates IRF3 and NF-κB, several noncanonical pathways have been reported. For example, STING/PERK/eIF2α signaling has been implicated in cell senescence (151), and in turn PERK may modulate the canonical pathway through binding to STING (39). The cGAS/STING pathway modulates autophagy, which contributes to innate immune response and metabolic homeostasis (152–155). Furthermore, STING has been reported to act as an ion channel that directly induces NLRP3 inflammasome activation (156). The importance of these noncanonical pathways to CVD pathophysiology remains to be elucidated.

Taken together, the cGAS/STING pathway is a promising therapeutic target for the treatment of CVDs. Indeed, as described above, in preclinical animal models, genetic and pharmacologic inhibition of the cGAS/STING pathway have protective effects against CVDs. However, nonspecific inhibition of this pathway might cause immune dysfunction and adverse effects, including impaired tissue repair. In addition, several studies have demonstrated crosstalk between the cGAS/STING and other signaling pathways (157), including TLR9 signaling (157–160), revealing complex and cell-type-specific relationships. Therefore, successful clinical translation of cGAS/STING-targeted therapies for CVD will require the development of strategies that enable spatiotemporal control of the cGAS/STING pathway.

## Conflict of interest

The authors have declared that no conflict of interest exists.

## Funding support

- Japan Science and Technology Agency research grant (Fusion Oriented REsearch for disruptive Science and Technology Program JPMJFR245G) to YH.
- Vehicle Racing Commemorative Foundation research grant to YH and MS.
- Takeda Science Foundation research grant to YH.
- SENSHIN Medical Research Foundation research grants to YH and DF.
- Moriya Scholarship Foundation research grant to YH.
- Koyanagi Foundation research grant to YH.
- JSPS KAKENHI grants JP23K07501 and JP26K02416 to YH, JP24K02451 to DF, and JP25K02646 to MS.
- Gout and uric acid foundation of Japan research grant to DF.
- BIOTRONIK Japan research grant to DF.

## Figures and Tables

**Table 2:**
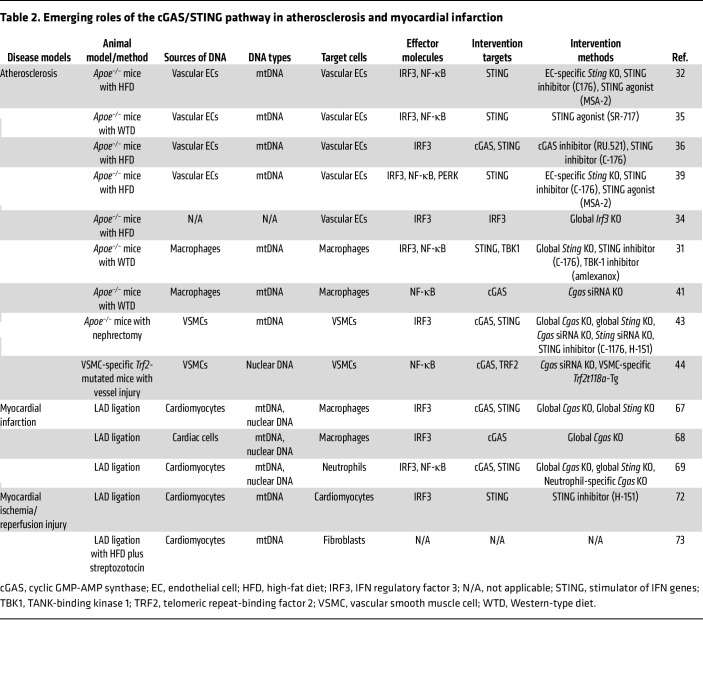
Emerging roles of the cGAS/STING pathway in atherosclerosis and myocardial infarction

**Table 3:**
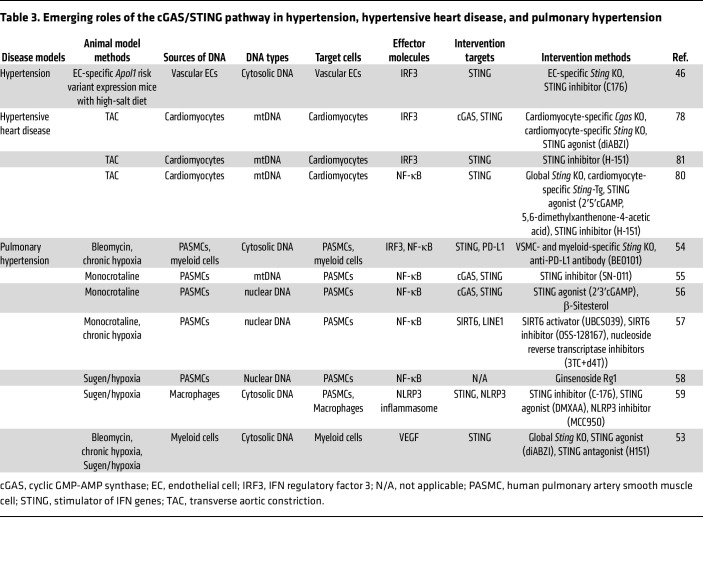
Emerging roles of the cGAS/STING pathway in hypertension, hypertensive heart disease, and pulmonary hypertension

**Table 1:**
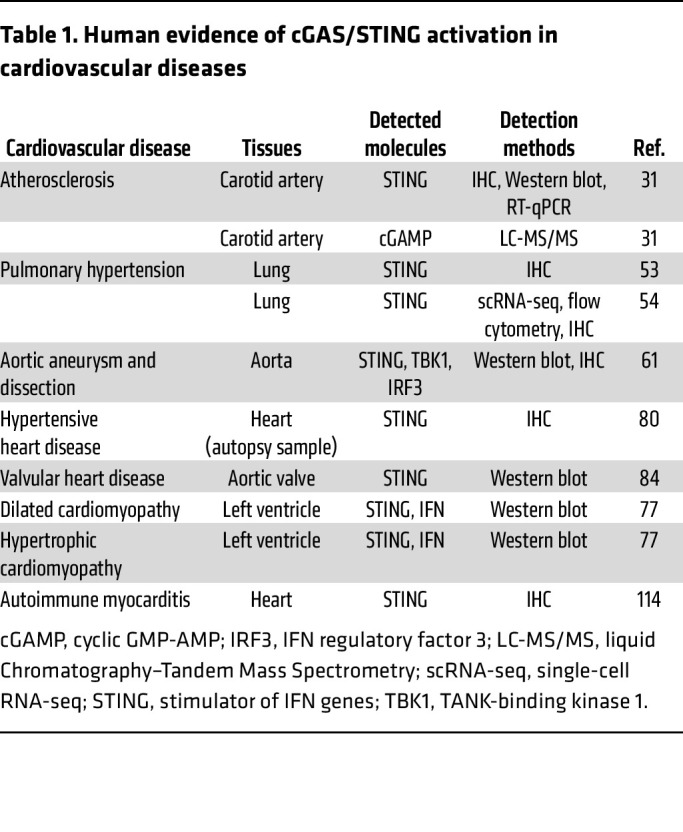
Human evidence of cGAS/STING activation in cardiovascular diseases

**Table 4:**
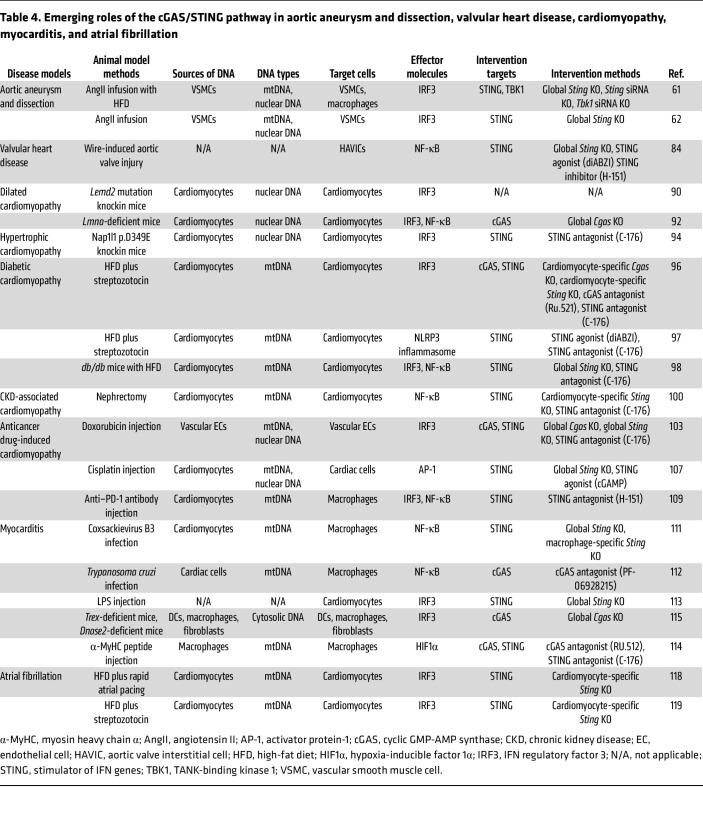
Emerging roles of the cGAS/STING pathway in aortic aneurysm and dissection, valvular heart disease, cardiomyopathy, myocarditis, and atrial fibrillation

**Table 5:**
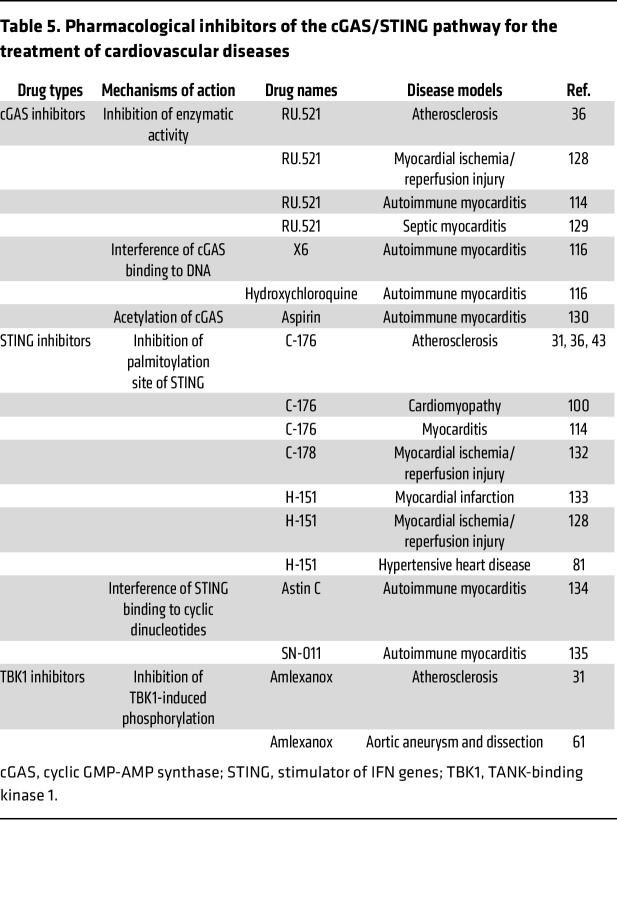
Pharmacological inhibitors of the cGAS/STING pathway for the treatment of cardiovascular diseases

## Version 1 07/01/2026

Electronic publication
